# »Dieser Fehler ist einzig und allein mein Fehler« – Politische Kommunikation im Zeichen der Corona-Pandemie

**DOI:** 10.1007/s41244-021-00207-1

**Published:** 2021-08-04

**Authors:** Constanze Spieß

**Affiliations:** Institut für Germanistische Linguistik, Philipps-Universität Marburg, Marburg, Deutschland

**Keywords:** Politische Rede, Ansprache, Regierungserklärung, Sprechhandlungen, Komplexe topische Muster, Metapher, Positionierungshandlungen, Political Speech, Address, Government Declarations, Speech Acts, Complex Topical Patterns, Metaphor, Practices of Positioning

## Abstract

In der aktuellen Corona-Pandemie stellt die Kommunikation politischer Akteur:innen an die Bevölkerung eine wichtige Maßnahme dar, um die politischen Entscheidungen zu begründen und zu legitimieren. Der vorliegende Beitrag untersucht die politische Kommunikation an die Bevölkerung am Beispiel der Ansprachen und Regierungserklärungen des österreichischen Bundeskanzlers Sebastian Kurz und der deutschen Bundeskanzlerin Angela Merkel im Zeitraum von März 2020 bis März 2021. Sind der Redeanlass und das intendierte Ziel der Ansprachen und Erklärungen der Akteur:innen typologisch gleich, zeigen sich in der konkreten sprachlichen Realisierung der Ansprachen und Erklärungen deutliche Unterschiede, aber auch Gemeinsamkeiten. Der Beitrag nimmt insbesondere die argumentative Struktur, die Redehandlungen, die damit verbundenen Positionierungs- und Adressierungsverfahren sowie die kommunikativen Strategien in den Blick und arbeitet sowohl Gemeinsamkeiten als auch Unterschiede der Reden heraus.

## Abstract (en)

In the current COVID pandemic, the communication by political actors addressing the public is an important means to justify and legitimize political decisions. This contribution examines political communication aimed at the public at the example of addresses and government declarations by Chancellor of Austria Sebastian Kurz and Chancellor of Germany Angela Merkel during March 2020 and March 2021. When occasion and the intended goal of the actors’ speeches are same, clear differences become visible in the concrete linguistic realization of the addresses and government declarations, yet also similarities can be seen. The contribution focuses particularly on the argumentative structure, speech acts and associated practices of positioning and addressing as well as communicative strategies in order to elaborate both similarities and differences between the speeches.

## Einleitung^1^

Zum Zeitpunkt des Abfassens dieses vorliegenden Beitrags herrscht die Pandemie seit mehr als einem Jahr auf der gesamten Welt und ein Ende ist vorerst noch nicht in Sicht, vielmehr hat die Pandemie trotz mittlerweile vorhandener Impfungen gegen das Virus die Welt fest im Griff. Die Pandemie stellt eine Herausforderung für das politische Handeln dar und schlägt sich sowohl im politischen wie auch gesellschaftlichen Bereich nieder. Der Sprache kommt dabei eine entscheidende Rolle zu, denn einschneidende soziale Ereignisse, politisches Handeln und staatliche Maßnahmen werden sprachlich vermittelt, bevor sie und indem sie in Kraft treten. Somit ist die Corona-Krise geprägt durch eine bis heute andauernde Krisenkommunikation.

Dass die Situation im März 2020 von der Politik als sehr ernst eingestuft wurde, war unter anderem daran zu erkennen, dass die deutsche Bundeskanzlerin Angela Merkel sich am Abend des 18.3.2020 in einer TV-Ansprache an die Bevölkerung Deutschlands zu Wort meldete. Bereits die Wahl des Kommunikationsformats, das Merkel ansonsten nur zum Jahreswechsel (und sonst nie) präferiert, sorgte nicht nur in den öffentlichen Medien für besondere Aufmerksamkeit, was sich unter anderem in der Diskussion über die TV-Ansprache der Kanzlerin sowohl im Vorfeld der Ansprache als auch danach medial niederschlug, sondern zeigte sich auch in den internetbasierten sozialen Netzwerken, auf denen die Rede positiv wie negativ kommentiert wurde.^2^

Ein paar Tage zuvor, am 15.3.2020, wandte sich der österreichische Bundeskanzler Sebastian Kurz ebenso in einer Fernseh-Ansprache an die »Österreicherinnen und Österreicher«^3^, um die bevorstehenden Maßnahmen zu verkünden^4^. Auch seine Rede wurde medial vielfach kommentiert und die Ansprache im TV galt ebenso als ungewöhnlich. Die Intentionen beider Reden waren gleich, ging es doch darum, die beschlossenen Maßnahmen im Zuge der Corona-Pandemie zu begründen und an die Bevölkerung zu appellieren, die Maßnahmen zu befolgen, damit sich die Ausbreitung des Virus verlangsamt. Trotz gleicher Intentionen weisen die Ansprachen und Erklärungen Sebastian Kurz und Angela Merkel als politische Akteur:innen aus, die unterschiedlich mit der Thematik umgehen, was sich sprachlich an verschiedenen Phänomenen manifestiert.

Beide Ansprachen gelten mittlerweile als historische Ansprachen an die Bevölkerung, die als besonders wahrgenommen wurden. Was im März 2020 noch niemand wusste, war, dass sich Ansprachen an die Bevölkerung und Regierungserklärungen im Kontext der Pandemie noch viele Male wiederholen würden, dass Angela Merkel und Sebastian Kurz bis heute immer wieder im Hinblick auf die Bewältigung der Pandemie sich an die Bevölkerung wenden sollten und die jeweils erste Ansprache der beiden dabei bis heute relevant ist.^5^

Im Zentrum der sogenannten Krisenkommunikation steht aber immer ein zentrales Ziel, um das die hier untersuchten Reden und Erklärungen kreisen: die Eindämmung der Virusaktivität und die Erlangung der Kontrolle über das Virusgeschehen. Verbunden damit sind natürlich viele weitere thematische Aspekte (z.B. die Debatte um psychische Folgen von Einsamkeit oder die wirtschaftlichen Folgen der Pandemie, die Situation auf den Intensivstationen, die Impfkampagne, gefährliche Virusmutationen etc.), die in den Reden mehr oder weniger ausgeführt oder auch nur angedeutet werden.

Der vorliegende Beitrag widmet sich der pandemiebedingten Krisenkommunikation von Angela Merkel und Sebastian Kurz im Zeitraum von März 2020 bis März 2021 und nimmt die Ansprachen in verschiedenen Medien und Regierungserklärungen im Parlament in den Blick. In einem ersten Schritt werden dazu in Abschnitt 2 die Textsorten bzw. Redegattungen zunächst kurz vorgestellt, um dann im Anschluss daran in Abschnitt 3 die realisierten sprachlichen Mittel, die die beiden politischen Akteure in den Reden verwenden, zu vergleichen und sowohl Gemeinsamkeiten als auch Unterschiede herauszuarbeiten. Im Fokus der Analyse stehen dabei die verwendeten topischen Muster, die Redehandlungen, Adressierungs- und Positionierungsphänomene sowie abschließend die Bestimmung der kommunikativen Redestrategien, die den Argumentationen zugrunde liegen.

## Die Redegattungen Ansprache und Regierungserklärung im Kontext der Corona-Pandemie

Um einschneidende Ereignisse im Kontext der Corona-Pandemie an die Bevölkerung zu kommunizieren, wählen Sebastian Kurz und Angela Merkel ähnliche, aber teils auch unterschiedliche Wege. So stellen für Sebastian Kurz die Pressekonferenzen ein zentrales Mittel der Kommunikation dar, Angela Merkel nimmt wöchentlich Kontakt zur Bevölkerung auf^6^, indem sie eine Videobotschaft bzw. einen Podcast verfasst, aber auch TV-Ansprachen oder Facebookposts^7^ sowie Pressekonferenzen werden genutzt, um die Entscheidungen der Regierung der Bevölkerung sowie der Medienöffentlichkeit zu kommunizieren.

TV-Ansprachen und Regierungserklärungen zu außergewöhnlichen Anlässen gehören zur Großgruppe der politischen Rede und stellen eine Spezialform dieser dar^8^. Sie dienen in erster Linie dazu, ein möglichst großes Publikum, die gesamte Bevölkerung des Landes, anzusprechen, die politischen Handlungen vor diesem Publikum zu legitimieren, Zustimmungsbereitschaft zu erzeugen und letztlich das Publikum zu gemeinschaftlichem Handeln zu bewegen. Dadurch, dass sie sich an die gesamte Bevölkerung eines Landes richten, also an ein disperses und verstreutes Publikum gerichtet sind, können sie als mehrfach adressiert gelten und im Hinblick auf die Kommunikation im Parlament auch als triadische Kommunikation beschrieben werden (vgl. Kühn 1995; Dieckmann 1985,9).

Auch wenn sich im Kontext der Corona-Kommunikation zentrale Funktionen, sprachliche Handlungen, topische Muster und Strategien bei den hier untersuchten Reden/Ansprachen gleichen, unterscheiden sie sich hinsichtlich der Lokalität, der Adressatenorientierung und auch im Hinblick auf die jeweilige sprachliche Gestaltung oder die inhaltliche Schwerpunktsetzung. Während TV-Ansprachen und Podcasts primär die Bevölkerung eines Landes adressieren, sind Regierungserklärungen gerahmt vom parlamentarischen Setting. Sie sind sozusagen eingebunden in eine parlamentarische Debatte und bilden in den hier untersuchten Exemplaren den Ausgangspunkt für die parlamentarischen Auseinandersetzungen um die Maßnahmen zur Pandemiebekämpfung. Dementsprechend sind sie nicht nur an die (mediale) Öffentlichkeit adressiert, sondern haben auch die an der Debatte teilnehmenden Akteur:innen, also die Parlamentarier:innen als zunächst primäre Adressat:innen vor Augen (vgl. Burkhardt 2003, 2005, 2017). Regierungserklärungen dienen dazu, Entscheidungen vor der Öffentlichkeit und der gegnerischen Partei zu erläutern und zu legitimieren. Sie gelten als Absichtsreden, die das Handeln bei einschneidenden Ereignissen begründen. Dementsprechend sind sie in ihrer dominanten Grundfunktion informativ-persuasiv ausgerichtet. Um von den Handlungen und Entscheidungen zu überzeugen, werden Argumente angeführt. Insofern handelt es ich auch um deutlich argumentativ strukturierte politische Reden. Stüwe (2005) bescheinigt der Regierungserklärung mehrere Funktionen. So sind Regierungserklärungen gekennzeichnet durch mindestens fünf Funktionen, die aber nicht immer in gleichem Maße realisiert sein müssen: die Informationsfunktion, die Appellfunktion, die Beziehungsfunktion (realisiert beispielsweise in den hier untersuchten Reden durch expressive Sprechhandlungen wie Dankesbekundungen), die Solidarisierungs- und Integrationsfunktion und die Funktion der Selbstdarstellung und Imagepflege (vgl. dazu Stüwe 2005, S. 153–242). Ergänzend ist hier hinzuzufügen, dass die Informations- und Appellfunktion auch als informativ-persuasive Funktion (vgl. Girnth 2015) erfasst werden kann. In den Funktionen lassen sich auch die von Klein (1998) und Efing (2005) herausgearbeiteten kommunikativen Strategien wiederfinden, auf welche im vorliegenden Beitrag abschließend (Kap. 4) eingegangen wird. Alle Funktionen der Regierungserklärung finden sich auch in den Ansprachen von Merkel und Kurz, wenngleich die informativ-persuasive Information dominant ist, da es um die Überzeugung der Bevölkerung geht, die politischen Maßnahmen der Pandemiebekämpfung mitzutragen.

## Befunde

### Zum Korpus

Untersuchungsgrundlage stellen 38 Texte dar, bestehend aus unterschiedlich umfangreichen Ansprachen, Regierungserklärungen und Pressestatements des österreichischen Bundeskanzlers Sebastian Kurz und der deutschen Bundeskanzlerin Angela Merkel, die in einem Zeitraum von März 2020 bis März 2021 gehalten wurden.^10^

Mit Beginn der Corona-Pandemie im März 2020 traten Angela Merkel und Sebastian Kurz vor die jeweilige Landesbevölkerung, um über die Maßnahmen, die die Ausbreitung der Pandemie verhindern sollten, zu informieren und diese schließlich auch zu legitimieren. Die in dem genannten Zeitraum realisierten Reden bzw. Ansprachen und Regierungserklärungen von Kurz und Merkel wurden im Hinblick auf topische Muster, Redehandlungen, Adressierungs- und Positionierungsphänomene sowie kommunikative Redestrategien untersucht. Im Folgenden wird der Frage nachgegangen, inwiefern sich die Ansprachen der politischen Akteur:innen unterscheiden und inwiefern sie Gemeinsamkeiten aufweisen, inwiefern sich aber auch die Ansprachen der einzelnen Akteur:innen im Laufe der Zeit hinsichtlich der eingesetzten sprachlichen Mittel verändert haben.

Zentrale Themen der untersuchten Texte waren bis Jahresende 2020 sowohl bei Merkel als auch bei Kurz die politischen Maßnahmen zur Eindämmung der Pandemie, insbesondere die Kontakteinschränkungen, Lockdown-Maßnahmen, aber auch Wirtschaftshilfen und Förderinstrumente. Mit Beginn des Jahres 2021 zeigt sich eine Themenverlagerung bzw. -erweiterung. So war zu Beginn des Jahres für Kurz vor allem die Ausweitung von Corona-Teststrategien ein zentrales Thema und seit Ende Februar bestimmt die Thematik des Impfens die Pressekonferenzen.^11^ Auch bei der deutschen Bundeskanzlerin zeigt sich in den Pressekonferenzen, Ansprachen und Regierungserklärungen seit Januar 2021 eine Fokussierung auf die Impfthematik und ab Februar gewinnt die Debatte um die Ausweitung von Teststrategien an Gewicht (vgl. Merkel 2021a, b, c, d, e).^12^

### »*Wir müssen jetzt alles tun, damit das Virus sich nicht unkontrolliert ausbreitet*.«^13^ – Begründungsmuster der Krisenkommunikation

Die Analyse der Ansprachen und Regierungserklärungen hat gezeigt, dass bestimmte Redehandlungen (wie z.B. *informieren, warnen, appellieren, feststellen, vermuten, versprechen*), Adressierungen, Selbstpositionierungen und Argumentationsmuster^14^ (topische Muster) in Zusammenhang mit positiv bewerteten lexikalischen Einheiten^15^ in allen Reden realisiert wurden und die sprachlichen Mittel unterschiedlicher Komplexitätsstufe im Dienst der komplexen sprachlichen Handlung des Argumentierens stehen.

Die untersuchten Ansprachen und Erklärungen haben sämtlich eine argumentative Grundstruktur. Dabei treten verschiedene Argumenttypen musterhaft auf, was Klein (2014, 2019) auch als topische Muster bezeichnet. In den untersuchten Texten geht es darum, die beschlossenen Maßnahmen zur Eindämmung der Pandemie zu legitimieren und zu begründen, von den Maßnahmen zu überzeugen, aber auch darum, Zusammenhänge zu erläutern und zu erklären sowie auf handlungsleitende Prinzipien zu verweisen (vgl. Klein 2019). Augenfällig in den hier untersuchten Ansprachen und Erklärungen ist, dass die Legitimation der Maßnahmen geprägt ist von einem Grundmuster des politischen Argumentierens, das aus einer Vernetzung verschiedener Argumenttypen besteht (vgl. hierzu Klein 2014, 2019), die die Argumente für das den Ansprachen und Erklärungen zugrunde liegende politische Handeln bzw. für politische Entscheidungen liefern. So werden die Reden und Ansprachen situativ gerahmt durch das Anführen von Situationsdaten, was Klein (2014, 2019) unter dem Terminus Datentopos fasst. In den untersuchten Ansprachen handelt es sich um die Schilderung der pandemischen Situation. Neben der Schilderung der Situation erfolgt eine Bewertung der Situation, die die zu begründenden Handlungen motiviert, was in den untersuchten Texten beispielsweise durch die Bewertung der pandemischen Situation als *ernst* erfolgt (vgl. Beleg 25). Klein (2014, 2019) spricht hier vom Valuationstopos. Bei der Handlungsbegründung wird Bezug genommen auf handlungs- und bewertungsleitende Prinzipien/Normen/Werte, was Klein als Prinzipientopos bezeichnet. Und ebenso bei allen hier untersuchten Texten werden die Ziele des Handelns benannt, was Klein als Finaltopos bestimmt. Neben diese »topische Basiskonstellation« (Klein 2019, S. 339) treten in den Reden noch weitere Argumentationsmuster hinzu. So ist der Verweis auf Handlungsfolgen oder auf Folgen des Nicht-Handelns als Konsequenzentopos beschreibbar. Der Autoritätstopos manifestiert sich sprachlich in den untersuchten Texten beispielsweise im Verweis auf Virolog:innen oder Epidemiolog:innen als wissenschaftliche Expert:innen. In den Ansprachen, die auf das Impfen verweisen, kommt zusätzlich der Realisierbarkeitstopos, der »die Realisierbarkeit des favorisierten Handelns« benennt (Klein 2019, S. 340), zur Geltung (vgl. Merkel c und e). Klein konstatiert, dass sich »[a]us den Argumenten, die sich auf diese Topoi verteilen, […] die Befürwortung des jeweiligen politischen Handelns als Schlussfolgerung [ergeben]« (Klein 2019, S. 338).

Das gemeinsame, feste Auftreten bestimmter Topoi in Form fester Verbindungen bezeichnet Klein (2019, S. 338) als »komplexe topische Muster«, die sich in vielen politischen Kommunikaten finden. In den vorliegenden Ansprachen und Reden gehen Datentopoi, Bewertungstopoi, Prinzipientopoi, Konsequenzentopoi und Finaltopoi Hand in Hand, so dass zumindest ausgesagt werden kann, dass diese Toposkategorien prägende Muster der Ansprachen und Erklärungen sind. Die Verbindung dieser Muster stellt somit eine Handlungsstruktur dar, die essentiell für die Begründung politischer Entscheidungen in der Corona-Pandemie in den hier untersuchten Ansprachen und Reden ist. So lässt sich die topologische Formation der Corona-Ansprachen in ihrer Grundstruktur (im Anschluss an und modifiziert nach Römer 2017, S, 155 f.) als ein Zusammenspiel spezifischer Topoi darstellen, die wiederum durch spezifische sprachliche Handlungen konstituiert werden und die die Argumente für die strittige These liefern (vgl. Tab. 1).

**Tab. 1:** Argumenttypen (modifiziert nach Klein 2003, 2014, 2019) in den Ansprachen und Erklärungen von Sebastian Kurz und Angela Merkel

**Argumenttyp**	**Erläuterung**	**These/Conclusio**
Datentopos	Im Datentopos werden Fakten/Tatsachen, Annahmen über die Situation angeführt, die ein bestimmtes politisches Ziel rechtfertigen	Umstrittene/zu begründende Politische Maßnahmen
Bewertungstopos^a^	Der Bewertungstopos nimmt eine Bewertung der Situationsannahmen/des als Faktum Geltenden vor und motiviert die politischen Handlungsziele	
Prinzipientopos	Der Prinzipientopos führt Prinzipien, Normen und Werte, die das Handeln leiten an.	
Finaltopos	Der Finaltopos benennt das Ziel	
(Konsequenzentopos)	Der Konsequenzentopos geht auf die Handlungsfolgen ein	
(Autoritätstopos)	Der Autoritätstopos führt als Argument Autoritäten an (z.B. Experten, im Falle des Corona-Diskurses unter anderem. Epidemiolog:innen, Virolog:innen)	

^a^Bei Klein (2003) wird dieser Topos noch als Motivationstopos benannt, später spricht Klein (2019) vom Valuationstopos. In diesem Beitrag wurde die Bezeichnung Bewertungstopos gewählt.

Klein (2003) erläutert die Argumenttypen folgendermaßen:

Politische Handlungen (Unterlassungen) werden begründet durch Ziele (Finaltopos), diese werden motiviert durch Situationsbewertungen (Motivationstopos), welchen wiederum einerseits bestimmte Annahmen über Situationsdaten (Datentopos) und oft auch über deren Konsequenzen (Konsequenztopos) und andererseits bewertungs- und handlungsleitende Prinzipien oder Werte (Prinzipientopos) zugrunde liegen. (Klein 2003, Sp. 1468)

Im Folgenden wird auf die Spezifik der in den untersuchten Reden immer wieder auftauchenden topischen Muster näher eingegangen.

#### Daten- und Bewertungstopos

Die Situierung in allen hier untersuchten Ansprachen und Erklärungen konstitutiert sich aus dem Bezug zur aktuellen Pandemiesituation, der wiederum aus einer Situationsdarstellung, einer Situationsdeutung und/oder einer Situationsbewertung bestehen kann. Diese Situierung kann Argumente für Handlungsbegründungen liefern. Die hier untersuchten Ansprachen und Regierungserklärungen sind ferner situativ gerahmt durch das jeweils besondere mediale Setting (TV, Video-Podcast, Facebook-Post oder Parlament).

In den Ansprachen vom März 2020 findet sich sowohl bei Merkel als auch bei Kurz ein Bezug zur Pandemiesituation, der aber unterschiedlich realisiert wird. Beide beginnen ihre Rede an die Bevölkerung zunächst mit einer knappen Situationsbewertung (vgl. Belege 1 und 2).

1. Wir erleben eine Zeit der Herausforderung, eine Zeit der Krise, in der es wichtig ist, dass wir zusammenstehen (Kurz 2020a, 15.3.2020)
2. … das Coronavirus verändert zurzeit das Leben in unserem Land dramatisch. Unsere Vorstellung von Normalität, von öffentlichem Leben, von sozialem Miteinander – all das wird auf die Probe gestellt wie nie zuvor. (Merkel 2020a, 18.3.2020)

Im Anschluss an den situationsbewertenden Einstieg werden Tatsachen/Fakten/Annahmen über die Situation angeführt, die die politischen Maßnahmen wie Kontakteinschränkungen, Schließung von Kindergarten, Kita, Schule, Handel und Gastronomie etc. rechtfertigen (vgl. Belege 3‑5). Das Anführen von Situationsdaten (Datentopos) und die Bewertung der Situation (Valuationstopos) ist somit eng miteinander verbunden und kann nicht immer strikt voneinander getrennt werden. Bei Merkel und Kurz erfolgt die jeweilige Begründung der Maßnahmen mithilfe des Bewertungstopos durch eine negative Situationsdarstellung, mithin durch die Benennung von Gefahren, durch eine metaphorische Bezugnahme auf Krieg^16^ oder durch die Feststellung, dass die Situation *ernst* sei (vgl. Beleg 25). Die zugrunde liegenden Topoi sind bei beiden typologisch gleich, die sprachliche Realisierung (und damit auch die Semantik) aber unterschiedlich (s.u.). Eine erste positive Situationsbewertung erfolgt nach dem ersten Lockdown im April unter anderem auch in Verbindung mit Emotionsbekundungen (vgl. Beleg 9). Die Situationsdeutung wechselt im Laufe der Zeit zwischen negativer und positiver Situationsbewertung und der entsprechenden Funktionalisierung von Emotionsvokabular je nach Verlauf der Pandemie.

Die Situationsbewertung (Belege 1 und 2) führt direkt in die Situationsdarstellung, bei der Kurz direkt auf das Nachbarland Italien verweist (Beleg 3), um mit der Darstellung der Situation in Italien implizit eine *Warnung* für Österreich bzw. an die österreichische Bevölkerung *auszusprechen*, aber zugleich auch um damit *hervorzuheben*, dass die Situation in Österreich besser ist als in Italien. Bei Merkel dagegen wird die aktuelle pandemische Situation konkretisiert, indem auf die Maßnahmen wie z.B. Schul- und Kitaschließungen Bezug genommen wird, wodurch Merkel die Bürger:innen als Betroffene situiert und positioniert (Beleg 4).

(3) Wir müssen uns vor Augen führen, dass in unserem Nachbarland Italien das Gesundheitssystem vor dem Zusammenbruch steht. […] Ich sage das nicht, um Sie in Panik zu versetzen. Sondern ich sage das, weil ich täglich noch mit Menschen konfrontiert bin, auch in Entscheidungspositionen, die versuchen die Situation zu verharmlosen. Noch haben wir die Chance, die Ausbreitung einzudämmen oder zumindest zu verlangsamen und somit Leben zu retten. (Kurz 2020a, 15.3.2020)
(4) Millionen von Ihnen können nicht zur Arbeit, ihre Kinder können nicht zur Schule oder in die Kita, Theater und Kinos und Geschäfte sind geschlossen, und, was vielleicht das Schwerste ist: uns allen fehlen die Begegnungen, die sonst selbstverständlich sind. Natürlich ist jeder von uns in solch einer Situation voller Fragen und voller Sorgen, wie es weitergeht. (Merkel 2020a, 18.3.2020)

Die Situationsdarstellung erfährt im Laufe der Rede immer wieder Konkretisierungen und kann als Rahmung der Rede gelten. Auch in den übrigen untersuchten Ansprachen und Reden findet jeweils eine Situationsdarstellung und -deutung sowie auch -bewertung statt, die Argumente für die beschlossenen Maßnahmen liefern.

(5) Guten Tag, ich melde mich zu meinem Podcast diesmal aus der häuslichen Quarantäne – deshalb auch am Telefon. Das ist ja eine Situation, die ich gerade mit vielen Menschen teile. (Merkel 2020b, 28.3.2020).
(6) Vielleicht am schwersten fällt uns allen das, was uns gegen das Virus am meisten hilft. Kontakte vermeiden, Abstand statt Nähe zu anderen Menschen. Das ändert alles in unserem privaten Leben und bei der Arbeit. Da geht es mir wie Ihnen. (Merkel 2021b, 30.1.2021)

Die Situationsdarstellung, -deutung und -bewertung findet in den Reden und Erklärungen von Kurz, wie bereits angedeutet, ebenfalls statt; auffällig ist hier jedoch der Bezug zur Situation in anderen europäischen Ländern bei gleichzeitigem Verweis darauf, dass Österreich die Situation besser in den Griff bekommen hat als die genannten Länder. Dabei wird mit der sprachlichen Aufwertung des eigenen Handelns zugleich eine Vorwurfs- und Unterstellungshandlung anderen Ländern gegenüber vollzogen (Beleg 8 und 9). Mit der kontrafaktischen Äußerung *Dann sieht man nämlich sehr schnell, wie die Situation wäre, wenn wir als Österreich nicht gehandelt hätten* (Beleg 9) wird anderen Ländern unterstellt, dass sie nicht angemessen gehandelt haben. Mit der Aussage wird ein Schlussprozess in Gang gesetzt. So wird vor allem durch die grammatische Konjunktivkonstruktion inferiert, dass die anderen europäischen Länder im Handeln versagt haben, indem eine kontrafaktische Situation für Österreich aufgerufen wird, die aber für andere europäische Länder als faktisch gilt (*wie die Situation wäre, wenn…*). Der Schlussprozess lautet*: Die Situation ist in Österreich nicht so, also hat Österreich richtig gehandelt und die anderen Länder haben nicht richtig gehandelt*. Diese Situationsdarstellung und -bewertung wird getragen von der Strategie der Aufwertung der Eigengruppe, die Österreich als handelnde Instanz ausweist, und der Abwertung des Handelns bzw. Nicht-Handelns der anderen europäischen Länder. Zur nationalen Vergleichsrhetorik passt auch die soziale Kategorisierung in *Österreicherinnen* und *Österreicher* innerhalb der Anreden (vgl. Beleg 7, 8, 33, vgl. auch Abschnitt 3.5 in diesem Beitrag).

(7) In den letzten 24 Stunden haben in Frankreich über 1.000 Menschen ihr Leben aufgrund des Coronavirus verloren. In den USA sind mittlerweile über 250.000 Menschen infiziert und in unserem Nachbarland, in Italien, gibt es mittlerweile in den letzten Wochen über 14.000 Menschen, die aufgrund des Coronavirus verstorben sind. Und das Schlimme an all diesen Zahlen ist, dass das in vielen Ländern erst der Anfang ist.
  Österreich ist bisher besser durch diese Krise gekommen als andere Länder und der Grund dafür sind Sie, sehr geehrte Damen und Herren Österreicher. Wir haben schneller und restriktiver reagiert als andere. Sie haben durch Ihr Verhalten Leben gerettet und dafür möchte ich heute einmal ein ganz, ganz großes Danke sagen! (Kurz 2020d, 3.4.2020)
(8) Mich erfüllt es auch mit Freude, wenn ich höre, über welche Themen hier gesprochen werden kann. Weil, das zeigt uns, dass wir die letzten Wochen einiges richtig gemacht haben dürften. Dass es eben ein Faktum ist, dass wir die Krise besser gemeistert haben als andere Staaten, und uns daher jetzt schon die Frage stellen können. »Wie fahren wir das Land wider hoch?« – ganz im Gegenteil zu anderen Ländern, die sich diese Fragen nicht stellen können. Wenn ich jetzt die Frage höre: War das alles wirklich notwendig – so viele sind ja gar nicht gestorben – dann bitte ich Sie, schon den Grundregeln der Mathematik zu folgen und bei allen, bei denen das nicht funktioniert, mache ich den Vorschlag, in andere Länder in Europa zu schauen. Nach Italien zu schauen, nach Frankreich zu schauen, nach Spanien zu schauen. Dann sieht man nämlich sehr schnell, wie die Situation wäre, wenn wir als Österreich nicht gehandelt hätten. (Kurz 2020e, 22.4.2020)

Bis zum Sommer 2020 ist diese Form der Situationsdarstellung unter Zuhilfenahme der Strategie der Eigenaufwertung und der Fremdabwertung in den Reden von Kurz zu finden. Mit dem erneuten starken Anstieg der Infektionszahlen im Herbst 2020 spitzt sich diese Auf- und Abwertungsstrategie im Rahmen einer Situationsbewertung mit nachfolgender Handlungsbegründung insofern zu, als die Verantwortung für das Infektionsgeschehen im eigenen Land in das Ausland verlegt wird (vgl. Beleg 9), genaugenommen werden die Menschen, die in Österreich leben und im Sommer in ihre Herkunftsländer gereist sind, für den Infektionsanstieg verantwortlich gemacht.

(9) Sehr geehrte Damen und Herren. Wir hatten im Sommer sehr, sehr niedrige Ansteckungszahlen nach dem Lockdown und haben dann durch Reiserückkehrer und insbesondere auch durch Menschen, die in ihren Herkunftsländern den Sommer verbracht haben, uns Ansteckungen wieder ins Land **hereingeschleppt**. Daher ist es notwendig, dass wir diesmal auf ein sehr konsequentes Grenzregime setzen, das verhindern soll, dass wir in Österreich zwar mit den Zahlen nach unten kommen uns aber dann durch Auslandsreisen in der Weihnachtszeit **das Virus wieder ins Land schleppen**. (Kurz, Pressekonferenz vom 2.12.2020, Hervorh. CS)

Durch die in der Proposition des ersten Satzes von Beleg 9 enthaltene Prädikation (*Menschen, die in ihren Herkunftsländern den Sommer verbracht haben, uns Ansteckungen wieder ins Land hereingeschleppt*) wird eine Schuldzuweisung formuliert, die pauschal auf eine bestimmte soziale Gruppe zielt, nämlich auf Menschen, die zwar in Österreich leben, aber dort nicht geboren sind bzw. eine Zuwanderungsgeschichte aufweisen.^17^

Zwar wird in den Ansprachen Merkels auch auf die Gefahr der Virusausbreitung durch zu viel Mobilität hingewiesen, eine Schuldzuweisung an eine bestimmte soziale Gruppe wird jedoch nicht formuliert. Dies erfolgt jedoch nicht im Rahmen der Situationsbewertung, sondern im Kontext der Erläuterung und Darstellung der Maßnahmen in Regierungserklärungen im Deutschen Bundestag (vgl. Belege 10 und 11).

(10) Die Kontaktbeschränkungen beziehen sich vor allen Dingen auf private Kontakte. In der Öffentlichkeit soll es in Zukunft nur noch die Begegnung von zwei Hausständen, maximal zehn Personen, geben. Private Kontakte sind insgesamt auf ein absolut notwendiges Minimum zu reduzieren. Auf nicht notwendige private Reisen ist zu verzichten, auch im Falle von Besuch bei Verwandten. (Merkel 2020h, 29.10.2020)
(11) Private Reisen und Besuche, auch von Verwandten, sind weiter zu unterlassen, und das Arbeiten im Homeoffice muss, wo immer möglich, durchgesetzt werden. (Merkel 2021c, 11.2.2021)

Auch Merkel verweist in Situationsdarstellungen auf andere europäische Länder, jedoch mit dem Ziel, das gemeinsame europäische Vorgehen bzw. das gemeinsame politische Handeln durch die Aussage *dass es gut ist, dass wir diese Europäische Union haben* positiv zu bewerten und das gemeinsame Handeln zu rechtfertigen (vgl. Beleg 12).

(12) Die Fallzahlen steigen europaweit wieder rapide an. Ich ermuntere durchaus alle, einmal zu schauen, was in unseren Nachbarländern so los ist, wenn wir über die Lage bei uns debattieren. Das zeigt, dass wir hier kein spezielles deutsches Phänomen beobachten, sondern dass wir doch sehr ähnliche Entwicklungen in ganz Europa haben. […] Bei allen Beschwerlichkeiten glaube ich, dass sich in der Pandemie wieder gezeigt hat, dass es gut ist, dass wir diese Europäische Union haben; denn wenn wir uns die protektionistischen Tendenzen und die Weltlage betrachten, dann, glaube ich, war es richtig, dass wir in der deutschen Ratspräsidentschaft im zweiten Halbjahr 2020 die Weichen gestellt haben für wichtige gemeinsame europäische Vorgehensweisen. (Merkel 2021e, f, 25.3.2021)

#### Prinzipientopos

Mit dem Prinzipientopos wird auf Normen, Prinzipien und Werte rekurriert, die das politische Handeln motivieren und begründen. In Merkels Reden fällt auf, dass sie in besonderer Weise auf das Spannungsverhältnis von Individuum und Gesellschaft und die gegenseitige Bedingtheit, auf das gegenseitige Angewiesensein eingeht und dies als Handlungsmotivation ihren Appellen an die Bevölkerung zugrunde legt (vgl. Belege 13 und 14).

(13) Alle zählen, es braucht unser aller Anstrengung. Das ist, was eine Epidemie uns zeigt: wie verwundbar wir alle sind, wie abhängig von dem rücksichtsvollen Verhalten anderer aber damit eben auch: wie wir durch gemeinsames Handeln uns schützen und gegenseitig stärken können. Es kommt auf jeden an. Wir sind nicht verdammt, die Ausbreitung des Virus passiv hinzunehmen. Wir haben ein Mittel dagegen: wir müssen aus Rücksicht voneinander Abstand halten. (Merkel 2020a, 18.3.2020)
(14) Wir alle zusammen, eine überwältigende Mehrheit der Menschen in unserem Land, haben uns von Vorsicht, Vernunft und Verantwortung für andere leiten lassen. (Merkel 2020d, 30.5.2020)

Der Bezug auf das Gemeinsame im Zusammenhang mit der Verantwortung des Einzelnen wird bei Kurz auch hergestellt (vgl. Beleg 15), aber nicht in der Intensität wie bei Merkel, was unter anderem in den Ansprachen vom März in der Verwendung der Pronomen *wir* und *uns* schon auf der Textoberfläche deutlich wird (vgl. Tab. 2).

(15) Wir stehen vor einer Aufgabe, bei der alle gemeinsam zusammenstehen müssen, wir stehen vor einer Aufgabe, bei der jeder eine Verantwortung hat, und wir stehen vor einer Aufgabe, bei der jeder seinen Beitrag leisten muss. (Kurz 2020a, 15.3.2020)

#### Finaltopos

Der Finaltopos benennt das Ziel der zu begründenden und zu legitimierenden politischen Maßnahmen, nämlich die Kontrolle über das Virus durch das Eindämmen des Virus zu erlangen (Beleg 16) oder die Wiedererlangung von Freiheit (Beleg 17). Beleg 17 stellt zudem durch den Bezug auf den Freiheitsbegriff eine Verknüpfung mit dem Prinzipientopos dar, insofern das Ziel zugleich ein Prinzip/einen Wert darstellt, auf das/den immer wieder Bezug genommen wird.^18^

(16) Wir müssen jetzt alles tun, damit das Virus sich nicht unkontrolliert ausbreitet. Dabei zählt jetzt jeder Tag. Dafür müssen die Kontaktpersonen jedes infizierten Menschen benachrichtigt werden, um die Ansteckungsketten zu unterbrechen. (Merkel 2020f, 17.10.2020)
(17) Ich bin mir vollkommen bewusst, dass all diese Einschränkungen schwerfallen, aber diese Einschränkungen sind notwendig, damit wir die Freiheit wiedererlangen, die wir gewohnt sind. All diese Einschränkungen sind notwendig, damit wir bald schon wieder das Leben führen können, das wir so lieben. Und all diese Einschränkungen sind notwendig, damit wir das Leben auch mit den Menschen führen können, die wir so lieben, und nicht Menschen ihr Leben verlieren, die es nicht verlieren müssten. (Kurz 2020d, 3.4.2020)

Im Dienste der zentralen Zielformulierungen (Eindämmung des Virusgeschehens) sind alle anderen topischen Muster und Redehandlungen zu sehen.

#### Konsequenzentopos

Der Konsequenzentopos wird angeführt, um auf positive wie negative Folgen der Einhaltung der politischen Maßnahmen hinzuweisen. So wird mit dem Konsequenzentopos auf Gefahren aufmerksam gemacht, wenn keine politischen Maßnahmen erfolgen oder die Maßnahmen nicht konsequent befolgt werden (Belege 18 und 19), aber es werden auch Erfolge, die aus einer konsequenten Umsetzung der Maßnahmen resultieren, benannt (Beleg 8).

(18) Aber damit das auch so bleibt, müssen wir gemeinsam das exponentielle Wachstum abflachen und bremsen, sonst haben wir im Dezember 6.000 Neuinfizierte am Tag und drei Wochen später doppelt so viele. (Kurz 2020h, 18.10.2020)
(19) Deutschland hat ein exzellentes Gesundheitssystem, vielleicht eines der besten der Welt. Das kann uns Zuversicht geben. Aber auch unsere Krankenhäuser wären völlig überfordert, wenn in kürzester Zeit zu viele Patienten eingeliefert würden, die einen schweren Verlauf der Coronainfektion erleiden. (Merkel 2020a, 15.3.2020)

#### Autoritätstopos

Auf Autoritäten wie Virolog:innen und Epidemiolog:innen wird verwiesen, um die Plausibilität der Maßnahmen wie Abstand halten, Maske tragen und Handhygiene zu begründen.

(20) Aber wir müssen jetzt noch weiter gehen: Die Wissenschaft sagt uns klar: Die Ausbreitung des Virus hängt direkt an der Zahl der Kontakte, der Begegnungen, die jeder von uns hat. Wenn jeder von uns seine Begegnungen außerhalb der eigenen Familie jetzt eine Zeitlang deutlich verringert, dann kann es gelingen, den Trend zu immer mehr Infektionen zu stoppen und umzukehren. (Merkel 2020f und g, 17.10.2020)

Dem Verweis auf die Autorität der Wissenschaft folgt in Beleg 20 direkt der Finaltopos, der noch einmal das Ziel benennt, die Infektionen zu stoppen, wobei der Appell, die Kontakte einzuschränken, die Erreichung des Ziels begründet, was sprachlich durch die Verwendung eines Konditionalgefüges realisiert wird.

### »Bleiben Sie zu Hause!«^19^ – Redehandlungen der Krisenkommunikation

Dem Redeanlass entsprechend finden sich bei Merkel und bei Kurz appellative, expressive und informative sprachliche Handlungen, die in ihrem Zusammenspiel die Rede konstituieren und letztlich im Dienst der Handlungslegitimation der Einschränkungen (Kita- und Schulschließungen, Homeoffice, keine Veranstaltungen etc.) stehen und Teil der jeweiligen Begründungsmuster sind. Besonders gewichtig sind die appellativen Redehandlungen, da sie das Ziel der Ansprachen unterstützen.

Informative Sprechhandlungen sind im Kontext der Situationsdarstellung relevant (vgl. Belege 21-23), sie fungieren aber auch innerhalb des Finaltopos in Beleg 24 als Argumente; der Finaltopos wird in diesem Beleg durch die Aussage *unser großes Ziel muss natürlich sein, das zu verhindern *realisiert, wobei mit der Pro-Form *das* auf die in den informativen Sprechhandlungen vorgebrachten Argumente rückverwiesen und so der Konnex hergestellt wird. Darüber hinaus bereiten informative Sprechhandlungen im Kontext der Situationsdarstellung Appellhandlungen wie *damit das auch so bleibt, müssen wir gemeinsam das exponentielle Wachstum abflachen und bremsen* vor (Beleg 18). Auch hier wird mittels der Pro-Form *das* auf die informativen Sprechhandlungen rückverwiesen.

(21) Die Zahlen steigen also europaweit wieder rapide an. Mehr als eine halbe Million Menschen haben in der Europäischen Union bislang ihr Leben verloren, und der wirtschaftliche Schaden ist immens. (Merkel 2021c, 11.2.2021)
(22) Ein wesentlicher Bestandteil für den von BioNTech und Pfizer entwickelten Corona-Impfstoff wird in Klosterneuburg hergestellt. Es freut mich, dass ein österreichisches Unternehmen damit entscheidend zur Eindämmung dieser weltweiten Pandemie beitragen wird. (Kurz, Presseaussendung, 27.11.2020)
(23) Wir müssen uns vor Augen führen, dass unser gesamtes Nachbarland Italien unter Quarantäne steht, dass es dort sterbende Menschen gibt, die sich von ihren Angehörigen nur noch telefonisch verabschieden können, weil die Ansteckungsgefahr zu groß ist, dass dort Ärzte entscheiden müssen, wen sie behandeln, weil die Kapazitäten in den Spitälern nicht mehr ausreichen, um alle zu behandeln, die Hilfe brauchen. (Kurz 2020a, 15.3.2020)
(24) Wir haben derzeit in Österreich rund 1.000 bis 1.500 Neuinfizierte pro Tag und wir haben die Situation, dass sich diese Zahl innerhalb von drei Wochen verdoppelt. Und unser großes Ziel muss natürlich sein, das zu verhindern. Zusammengefasst heißt das: Alle, die sagen, wir haben derzeit kein Problem auf der Intensivmedizin, die haben vollkommen recht. Aber damit das auch so bleibt, müssen wir gemeinsam das exponentielle Wachstum abflachen und bremsen, sonst haben wir im Dezember 6.000 Neuinfizierte am Tag und drei Wochen später doppelt so viele. (Kurz 2020h, 18.10.2020)

Die informativen Sprechhandlungen im Kontext der Situationsdarstellung dienen der Vorbereitung der appellativen Sprechhandlungen und gleichzeitig geben sie Argumente zur Begründung der Maßnahmen. Sie sind vor dem Hintergrund des Final- und Konsequenzentopos zu sehen, die auf das Ziel der zu begründenden Handlungen wie auch auf die Konsequenzen des Handelns verweisen.

Im Zentrum der Ansprachen und Erklärungen stehen Appellhandlungen, die unter anderem durch Imperative (Beleg 25), aber auch durch Bitten (Beleg 26) realisiert werden, also mal als explizite (Beleg 25), mal als indirekte Sprechakte (Belege 26, 27) formuliert werden. Die Appellhandlungen stehen sämtlich im Dienst des Finaltopos und des Konsequenzentopos (z.B. Belege 16, 17 und 27).

(25) Es ist ernst. Nehmen Sie es auch ernst. (Merkel 2020a, 18.3.2020)
(26) Und deswegen möchte ich Sie heute noch einmal eindringlich bitten: Halten Sie sich weiter streng an diese Regeln. Verzichten Sie weitest möglich auf Kontakte außerhalb des Kreises der Menschen, mit denen Sie zusammenwohnen […]. Halten Sie Abstand. (Merkel 2020b 28.3.2020)
(27) Aber alles, was Menschen gefährden könnte, alles, was dem Einzelnen, aber auch der Gemeinschaft schaden könnte, das müssen wir jetzt reduzieren (Merkel 2020a, 18.3.2020)

Die Appellhandlungen erfolgen bei Angela Merkel einerseits durch die Hervorhebung des individuellen Handelns und der Hervorhebung der wichtigen Rolle jedes Einzelnen im Hinblick auf seine Verantwortung für die Gemeinschaft/Gesellschaft/das kollektive Ganze. Lexikalische Einheiten wie *wir, jede und jeder, Gemeinschaft, zusammen, unser, uns, gemeinsam, gegenseitig, einander, miteinander, beistehen* verweisen somit auf das Spannungsfeld von Individuum und Gesellschaft. Andererseits formuliert Merkel die Appelle bzw. Aufforderungen mit einem inkludierenden *wir müssen*. Sprachlich zeigt sich hier in der jeweils ersten Ansprache ein Unterschied zwischen den Ansprachen von Sebastian Kurz und Angela Merkel in der Verwendung der lexikalischen Einheiten, die die Appellfunktion unterstützen (vgl. Tab. 2).

**Tab. 2:** Vorkommenshäufigkeiten lexikalischer Einheiten in den Ansprachen vom 15.3.2020 (Kurz) und 18.3.2020 (Merkel)

**Ausdrucksmittel**	**Merkel**^**a**^		**Kurz**	
	*p*. tausend Wörter	absolut	*p*. tausend Wörter	absolut
*jede*	8	13	2	3
*wir*	24	40	12,4	18
*uns*	14,8	24	11	16
*gemeinsam*	1,8	3	2	4
*gegenseitig*	0,6	1	0	0
*miteinander*	1,2	2	0	0
*zusammen*	0,6	1	2	3
*Gemeinschaft*	2,5	4	0	0
solidar*	1,2	2	0	0
*wir müssen*	4,3	7	2	3
*beistehen*	0,6	1	0	0

^**a**^Der Wert bezieht sich auf die Verwendung der Ausdruckseinheit pro tausend Wörter. Die absoluten Zahlen stehen in der jeweils rechten Spalte. Der *Asterisk* gibt an, dass alle Ausdrücke mit dem Wortstamm *solidar* gezählt wurden.

Das verwendete Vokabular, das auf das Spannungsverhältnis von Individuum und Gesellschaft verweist, hat einen wesentlichen Anteil an der in den Ansprachen realisierten Persuasionsfunktion einerseits. Andererseits wird damit auch eine Solidarisierungs- und Integrationsfunktion realisiert, die im Dienst der Legitimation der pandemiebedingten Einschränkungen steht. Ziel der Reden ist es ja, Gemeinschaft zu erzeugen, um die Pandemie unter Kontrolle zu bringen bzw. die Ausbreitung des Virus zu verhindern und möglichst viele Bürgerinnen und Bürger davon zu überzeugen und für die veranlassten Maßnahmen einzunehmen. Aus diesem Grund erfolgt die besondere Hervorhebung der Rolle des Einzelnen für die Gemeinschaft.

(28) Dazu möchte ich Sie und uns alle gerade heute – zum 1. Advent und zu Beginn der Weihnachtszeit – einmal mehr ermutigen. Zeigen wir Menschen weiter, was in uns steckt, indem wir uns auch jetzt – im Winter, vor Weihnachten, zum Jahreswechsel – an die Regeln halten, die für uns alle gelten. Weil wir erleben werden, dass es sich lohnen wird. Weil wir so gemeinsam stärker sein werden als das Virus. (Merkel 2020k, 28.11.2020)

Dadurch dass sich die politischen Akteur:innen mit der Verwendung der Pronomen *wir* und *uns* oder des hier als Adverb genutzten Adjektivs *gemeinsam* (Beleg 28) mehr oder weniger als Teil der Bevölkerung positionieren, möchten sie von der Relevanz der Maßnahmen überzeugen. Die mit dem verwendeten Vokabular (vgl. Tab. 2) zugleich realisierte Solidarisierungs- und Integrationsfunktion steht damit im Dienst der Persuasion. Besonders deutlich zeigt sich das auch durch die Verwendung von *wir müssen*. Die Appelle Merkels und von Kurz, die Maßnahmen zu befolgen, werden unter anderem mit einem inkludierenden *wir* in Kombination mit dem Verb *müssen* realisiert (Beleg 29 und 30), das in seiner kontextuellen Semantik auf die Notwendigkeit der Maßnahmen oder gar auf einen äußeren Zwang verweist.^20^

(29) Aber alles, was Menschen gefährden könnte, alles, was dem Einzelnen, aber auch der Gemeinschaft schaden könnte, das müssen wir jetzt reduzieren. Wir müssen das Risiko, dass der eine den anderen ansteckt, so begrenzen, wie wir nur können. (Merkel 2020a, 18.3.2020)
(30) Wir haben ein Mittel dagegen: Wir müssen aus Rücksicht voneinander Abstand halten. Der Rat der Virologen ist ja eindeutig: Kein Handschlag mehr, gründlich und oft die Hände waschen, mindestens eineinhalb Meter Abstand zum Nächsten und am besten kaum noch Kontakte zu den ganz Alten, weil sie eben besonders gefährdet sind. (Merkel 2020a, 18.3.2020)
(31) Meine große Bitte an alle Menschen im Land lautet weiterhin: Bitte halten Sie sich an die Maßnahmen, auch wenn es schwerfällt. Wir müssen weiter durchhalten, denn nur so können wir die Ansteckungszahlen auf ein Niveau senken, das uns Öffnungsschritte ermöglicht. (Kurz, Pressestatement vom 27.11.2020)
(32) Deshalb müssen wir auf unserem Weg durch die nächsten Wochen vorsichtig und behutsam handeln. Noch sind wir nicht so weit, Kitas und Schulen wieder öffnen zu können. Je konsequenter wir uns jetzt verhalten, auf Kontakte verzichten und da wo sie unumgänglich sind, Abstand halten, Hygieneregeln beachten und Masken tragen, desto schneller wird das wieder möglich sein. (Merkel 2021b, 30.1.2021)

Die Verwendung von *wir müssen* erfolgt im Rahmen von Appellhandlungen im Zusammenhang mit verschiedenen Topoi, unter anderem einem Autoritätstopos (Beleg 30) sowie dem Finaltopos (Beleg 31), der das Ziel der Maßnahmen nennt oder der in Verbindung mit dem Konsequenzentopos (Beleg 32), der auf Folgen aufmerksam macht, realisiert wird. Mit dem Autoritätstopos (Beleg 30) wird ein Bezug zu Experten hergestellt, der das Argument zur These *Die Ausbreitung des Virus muss eingedämmt werden* darstellt.

Appellative Sprechhandlungen finden sich in allen Reden. Die Ansprache von Merkel vom 24.3.3021, in der sie einen Fehler im Corona-Management eingesteht, weicht insofern von anderen Reden ab, als sie hier in der 1. Person spricht, wenn sie eingesteht: »Dieser Fehler ist einzig und allein mein Fehler, denn am Ende trage ich für alles die letzte Verantwortung. Qua Amt ist das so, also auch für die am Montag getroffene Entscheidung zur sogenannten Osterruhe.« (Merkel, 24.3.2021) Die Handlung des Fehlereingestehens steht im Dienst der Strategie, glaubwürdig zu sein. Erst am Ende der Rede wechselt Merkel wieder im Kontext des Finaltopos zum inkludierenden *wir*.

Neben appellativen Handlungen spielen innerhalb der Ansprachen und Erklärungen auch expressive Sprechakte eine Rolle, mit denen ein psychisches Befinden der Sprecher:innen zum Ausdruck gebracht wird, was unter anderem im Rahmen von Situationsbewertungen (also im Kontext des Bewertungstopos) erfolgt. Es handelt sich dabei um Dankesbekundungen und den Ausdruck von Freude angesichts sinkender Infektionszahlen. Die Belege zeigen zudem, dass die expressiven Sprechhandlungen nicht isoliert realisiert werden, sondern im Konnex mit anderen sprachlichen Handlungen stehen, unter anderem mit appellativen Handlungen (vgl. Beleg 33) oder mit Feststellungen, die die Situation positiv werten (Beleg 34) sowie im Kontext des Bezugs auf handlungsleitende Normen/Prinzipien wie Vorsicht, Vernunft und Verantwortung (Beleg 35), die zum Handeln motivieren und das Ergebnis begründen.

(33) Und dafür möchte ich auch Ihnen, sehr geehrte Österreicherinnen und Österreicher, heute noch einmal ganz herzlich danken und ich verbinde es mit einer Bitte, nämlich: Seien wir auch diesen Herbst und Winter so diszipliniert wie möglich. Wenn wir die Grundregeln einhalten, insbesondere das Abstand halten, dann können wir auch diese herausfordernden Monate, die vor uns liegen, gut überstehen. (Kurz 2020g, 28.8.2020)
(34) Ich muss zugeben, ich verspüre ein Gefühl der Freude und der Erleichterung, wenn ich der Debatte hier im Parlament zuhören darf. Ich verspüre ein Gefühl der Freude und der Erleichterung deshalb, weil die Debatte, wie sie stattfindet, wieder sehr viel von Normalität hat, und sie zeigt auch, dass wir uns in Österreich andere Sorgen machen können, als andere Länder. Wenn ich höre: Wann sperrt die Schule auf? Wann welche Klasse? Wie ist das mit den Hygienemaßnahmen dort genau? Und da ein irrsinniger Druck darauf ist, jetzt schnell Antworten zu bekommen: Dann erfüllt mich das mit Freude, weil in unserem Nachbarland Italien werden die Schulen dieses Semester gar nicht mehr geöffnet. (Kurz 2020e, 22.4.2020)
(35) Was wir sagen können, und dafür bin ich unglaublich dankbar: Wir haben diese Prüfung bisher recht gut bestanden. Wir alle zusammen, eine überwältigende Mehrheit der Menschen in unserem Land, haben uns von Vorsicht, Vernunft und Verantwortung für andere leiten lassen. Und so haben wir viel geschafft in diesen vier Monaten. Ich könnte auch sagen: Wir haben uns viel erspart. Unsere gemeinsame Leistung ist nämlich das, was bei uns glücklicherweise nicht eingetreten ist. (Merkel 2020d, 30.5.2020)
(36) Ich versichere Ihnen: In der Bundesregierung sind wir uns sehr bewusst, wie hart der Alltag für viele Eltern und Kinder zurzeit ist. Das unterschätzt niemand von uns. Deswegen ist es mir wichtig, Danke zu sagen. Danke an alle, die diese Zeit der Herausforderungen und Zumutungen mit großem Einsatz und viel Geduld meistern. (Merkel 2021b, 30.1.2021)

Als Repräsentant:innen der Regierung formulieren Kurz und Merkel auch kommissive Sprechhandlungen, die die Regierung auf Handlungen festlegen bzw. in denen die Regierung den Bürgerinnen und Bürgern ein Versprechen oder eine Garantie gibt (Beleg 36). So wird in Beleg 37 explizit durch die performative Formel *Lassen Sie mich versichern*, eine Garantiehandlung vollzogen, die die Notwendigkeit der Maßnahmen unterstreicht. In Beleg 38 wird durch Kurz die Verpflichtungshandlung im Zusammenhang mit einer Appellhandlung formuliert.

(37) Lassen Sie mich versichern: Für jemandem wie mich, für die Reise- und Bewegungsfreiheit ein schwer erkämpftes Recht waren, sind solche Einschränkungen nur in der absoluten Notwendigkeit zu rechtfertigen. Sie sollten in einer Demokratie nie leichtfertig und nur temporär beschlossen werden – aber sie sind im Moment unverzichtbar, um Leben zu retten. Deswegen sind seit Anfang der Woche die verschärften Grenzkontrollen und Einreisebeschränkungen zu einigen unserer wichtigsten Nachbarländer in Kraft. […] Ich versichere Ihnen: Die Bundesregierung tut alles, was sie kann, um die wirtschaftlichen Auswirkungen abzufedern – und vor allem um Arbeitsplätze zu bewahren; Wir können und werden alles einsetzen … (Merkel 2020a, 18.3.2020)
(38) Ich verspreche Ihnen, dass wir als Bundesregierung alles Menschenmögliche tun werden, damit wir gut durch diese Krise kommen und ich verspreche Ihnen auch, dass wir alles Menschenmögliche tun werden, dass wir schnell wieder aus dieser Krise herauskommen. Daher bitte ich Sie alle, beobachten wir gemeinsam in den nächsten Tagen die Zahlen. Ziehen wir keine voreiligen Schlüsse aufgrund einiger positiver Signale. Es braucht nicht nur einen Trend, es braucht eine nachhaltige positive Entwicklung. (Kurz 2020d, 3.4.2020)

### »Liebe Österreicherinnen und Österreicher«^21^ – Adressierung und Positionierung in der Krisenkommunikation

Die Orientierung an den Adressat:innen stellt im Bereich der politischen Kommunikation eine wesentliche Strategie dar, der in allen Kommunikationsformen Rechnung getragen wird. Wen die Sprecher:innen adressieren, hat Auswirkung auf die Versprachlichung der Kommunikationsziele. Nicht immer sind Adressatenorientierungen aber so explizit, wie dies in den Ansprachen oder Regierungserklärungen der Fall ist.

Merkels Ansprachen sind an die Mitbürgerinnen und Mitbürger adressiert. Mit dieser Adressierung konzeptualisiert sich Merkel als eine Person aus der Bevölkerung und nicht ausschließlich als Repräsentantin eines politischen Amtes. Unterstrichen wird diese Positionierung dadurch, dass sie hervorhebt, gleiche oder ähnliche Erfahrungen wie viele Mitbürger:innen (Beleg 5) gemacht zu haben. Und ebenso betont sie, dass sie sich – wie viele andere auch – an bestimmten Prinzipien orientiert (Beleg 6), die der Pandemiebekämpfung dienlich sind.

Adressiert wird bei Merkel zudem jeweils die gesamte Bevölkerung des Landes, da die Maßnahmen alle im Land lebenden Personen betreffen. Deutlich wird das in der TV-Ansprache durch die Adressierung »Liebe Mitbürgerinnen und liebe Mitbürger« (Merkel 2020a, 18.3.2020). In den Podcasts wechselt Merkel dann zwischen der Anrede »Guten Tag« (Merkel 2020b 28.3.2020), »Liebe Mitbürgerinnen und Mitbürger« (Merkel 2020e, 20.6.2020; Merkel 2020f, 17.10.2020 und Merkel 2020g, 24.10.2020) oder gar keiner direkten Anrede. In den Regierungserklärungen adressiert sie direkt mit der Anrede »Sehr geehrter Herr Präsident! Liebe Kolleginnen und Kollegen!« (Merkel 2021e, 25.3.2021), indirekt ist hier natürlich auch die massenmediale Öffentlichkeit und damit auch die Bevölkerung im Sinne der triadischen Kommunikation (vgl. Kühn 1995; vgl. Dieckmann 1985; vgl. Fußnote 9) adressiert.

Die Ansprachen von Kanzler Kurz sind auch an die gesamte Bevölkerung gerichtet, explizit adressiert werden bis zum Herbst jedoch ausschließlich die Österreicherinnen und Österreicher, wenn es sich um TV-Ansprachen oder Ansprachen per Facebook handelt. Im Nationalrat wählt Kurz die Adressierung *Sehr geehrte Damen und Herren! Liebe Österreicherinnen und Österreicher!*

Die Adressierung an die Österreicherinnen und Österreicher zieht sich durch alle Ansprachen. Mit der Anrede werden jedoch jene Bevölkerungsgruppen ausgeschlossen, die keine Österreicherinnen und Österreicher sind, aber dennoch in Österreich leben. Das irritiert etwas, da es doch in den Ansprachen darum geht, alle in Österreich lebenden Menschen von den beschlossenen Maßnahmen zur Eindämmung der Pandemie zu überzeugen. Insbesondere Personen mit Zuwanderungsgeschichte, die in Österreich leben, aber keine österreichische Staatsbürgerschaft besitzen, werden durch diese Adressierung ausgeschlossen und ausgegrenzt. Die Adressierung bedient somit eine nationalistische Ideologie, die auf Kosten von Menschen mit Zuwanderungsgeschichte erfolgt.

Darüber hinaus finden sich konkrete Adressierungen von relevanten Berufsgruppen in allen untersuchten Ansprachen und Reden. Durch die Adressierung wird implizit deutlich, wie Merkel und Kurz sich selbst positionieren, Merkel als Mitbürgerin ohne Thematisierung von Herkunft, Kurz als Österreicher. Die Selbstpositionierung Merkels findet ihre Entsprechung auch vor allem in der in der März-Ansprache häufigeren Verwendung des Pronomens *wir*, das in den Ansprachen inkludierend verwendet wird, wenn es um die Formulierung von Appellen geht (vgl. Belege 28 und 29), während Kurz zu Beginn der Pandemie in seiner ersten Ansprache mit dem Pronomen *wir* häufig^22^ auf die Regierung Österreichs, deren Teil er ist, verweist (Beleg 39).

(39) Wir alle in der Bundesregierung sind uns dessen bewusst, dass das massive Einschränkungen für jeden Einzelnen sind, aber diese Schritte sind notwendig, um die Gesundheit der österreichischen Bevölkerung zu verteidigen und um insbesondere die ältere Generation in unserem Land zu schützen. (Kurz 2020a, 15.3.2020)

### »Österreich ist bisher besser durch diese Krise gekommen als andere Länder«^23^ oder »Es geht um den Zusammenhalt und die Solidarität in unserer Gesellschaft und in Europa«^24^ – Ein Fazit

Auf den ersten Blick weisen die untersuchten Reden und Erklärungen hinsichtlich des Gegenstandes und der Struktur große Ähnlichkeiten auf. So ist für die meisten Reden ein Zusammenspiel bestimmter topischer Muster zur Handlungsbegründung zu konstatieren. Bei genauerem Hinsehen zeigen sich jedoch Unterschiede in der konkreten sprachlichen Ausgestaltung, die letztlich auch eine unterschiedliche Realisierung für die im Bereich der Politik typischen sprachlichen Strategien bewirkt. So konstituieren die im vorliegenden Beitrag analysierten sprachlichen Phänomene in ihrem Zusammenspiel die Profilierungs‑, Prolongierungs-, die Aufwertungs- und Abwertungsstrategie sowie die Ausgrenzungsstrategie.^25^

Bei beiden politischen Akteur:innen kommen die Profiliierungsstrategie, die Prolongierungsstrategie und die Aufwertungsstrategie vor. Die Profilierungsstrategie zeigt sich unter anderem darin, dass sich Kurz und Merkel als aktiv in der Bewältigung der Krise darstellen und sich zugleich als um die Sorgen und Nöte der Bevölkerung Wissende positionieren. Die Prolongierungsstrategie kommt in den Ansprachen dadurch zur Geltung, dass die getroffenen Maßnahmen als zielführend und positiv im Rahmen der Pandemiebekämpfung beschrieben werden, indem sie im Kontext der Pandemie auf bislang Erreichtes verweisen und zugleich einen Ausblick in die Zukunft geben (siehe Beleg 19: *Deutschland hat ein exzellentes Gesundheitssystem, vielleicht eines der besten der Welt. Das kann uns Zuversicht geben* oder Beleg 7: *Österreich ist bisher besser durch diese Krise gekommen*). In Verbindung mit einer Appellhandlung fungiert der Verweis auf das bereits Erreichte als Argument zur Begründung des Handlungsziels. Sowohl Merkel als auch Kurz verweisen auf »Kontinuität als zukunftssicherndem Element« und insinuieren eine »lineare[…] (positive)[…] Fortentwicklung« (Efing 2005, S. 229) durch die Regierung in der Pandemiezeit. Eng damit verbunden ist die Profilierungsstrategie, die in den untersuchten Reden auf die Kompetenz der Regierungsparteien/der Regierung verweist.

Bei Kurz kommt zu den beiden Strategien jedoch noch die Abwertungsstrategie und die Ausgrenzungsstrategie gegenüber Nicht-Österreicher:innen hinzu, die sich vor allem im Kontext der Situationsdarstellung und -bewertung manifestiert (vgl. Belege 7 und 8). Kurz profiliert und prolongiert die Regierungspolitik mithin somit durch die Abwertung der Politik anderer europäischer Länder, während Merkel das politische Handeln Deutschlands im Hinblick auf die Pandemiebekämpfung in den Zusammenhang des europäischen Handelns stellt.

## Footnotes

1 Das titelgebende Zitat stammt aus der öffentlichen Entschuldigung Angela Merkels vom 24.3.2021, in der sie die Beschlüsse zur Osterruhe gekippt und sich für das ›Hin und Her‹ beim Volk entschuldigt hat. Online unter: https://www.sueddeutsche.de/politik/merkel-entschuldigung-osterruhe-wortlaut-1.5245458 sowie unter https://www.bundeskanzlerin.de/bkin-de/mediathek/merkel-statement-osterruhe-1881092!mediathek?query= (beides zuletzt aufgerufen am 1.4.2021).

2 So titelt beispielsweise ein Beitrag im Spiegel online vom 18.3.2021: »Hat Merkel in der Coronapolitik versagt? Ein Jahr nach der historischen TV-Ansprache« (https://www.spiegel.de/politik/deutschland/merkels-corona-politik-ein-jahr-nach-der-historischen-tv-ansprache-podcast-spiegel-daily-a-ff497a38-cea1-4461-b1e1-c7bd9c929c47) (zuletzt aufgerufen am 18.3.2021).

3 Kurz adressierte in seiner Rede: Sehr geehrte Damen und Herren, liebe Österreicherinnen und Österreicher! (vgl. Kurz 2020c).

4 Die deutsche Bundeskanzlerin Angela Merkel und der österreichische Bundeskanzler Sebastian Kurz haben das gleiche politische Amt inne und somit ist davon auszugehen, dass es ähnliche kommunikative Amtshandlungen gibt. Die beiden Länder Österreich und Deutschland gleichen sich zudem hinsichtlich des politischen und des sozialstaatlichen Systems. Das begründet, neben dem Gebrauch der deutschen Sprache, eine Vergleichbarkeit der Ansprachen hinsichtlich ihrer konkreten sprachlichen Ausprägungen. Der Vergleich wird zudem von der Annahme geleitet, dass – trotz formaler Ähnlichkeiten der beiden Länder und der gemeinsamen Landessprache– sich unterschiedliche Mentalitäten einerseits an der sprachlichen Oberfläche und andererseits in der semantischen Tiefenstruktur (beispielsweise durch Topoi und Metaphern) manifestieren.

5 Diese Relevanz zeigt sich unter anderem in der öffentlichen Thematisierung der Rolle der Ansprache, aber auch in den Rückbezügen der Protagonist:innen auf ihre ersten Ansprachen. Vgl. zur öffentlichen Thematisierung der Relevanz: (https://www.spiegel.de/politik/deutschland/merkels-corona-politik-ein-jahr-nach-der-historischen-tv-ansprache-podcast-spiegel-daily-a-ff497a38-cea1-4461-b1e1-c7bd9c929c47) (zuletzt aufgerufen am 18.3.2021).

6 In den wöchentlichen Ansprachen per Videobotschaft ist aber nicht jede Woche Corona das Thema gewesen. Vgl. dazu die Zusammenstellung der Videobotschaften von Angela Merkel unter: https://www.bundeskanzlerin.de/bkin-de/mediathek (zuletzt aufgerufen am 1.4.2021).

7 Die Ansprachen wurden teilweise von Sebastian Kurz auf Facebook gepostet und/oder im TV und/oder Youtube-Kanal ausgestrahlt.

8 Neben Ansprachen und Regierungserklärungen gibt es zahlreiche politische Redegattungen, die sich jeweils in ihrer Funktionalität, im Adressat:innenkreis, in der institutionellen Anbindung oder hinsichtlich des Redeanlasses unterscheiden, wie z.B. die Debattenrede (vgl. Burkhardt 2003, 2005, 2017), die Gedenk- und Erinnerungsrede (vgl. Schöpe 2019), die Parteitags- und Beratungsrede (vgl. Bock 2019), die Wahlkampfrede (vgl. Spieß 2019), die Rück- und Antrittsrede (vgl. Müller/Recknagel 2019) oder die Aschermittwochsrede, um nur einige Redesorten zu erwähnen. Einen Überblick über die Differenzierung politischer Textsorten und Redegattungen gibt auch Klein (2000).

9 Dieckmann (1985) spricht hier auch von triadischer Kommunikation, bei der es zwar primär Adressierte bzw. primär Angesprochene gibt, aber immer auch sogenannte ›dritte‹, nicht primär adressierte Zuhörende, aber primär gemeinte Adressat:innen; diese triadische Konstellation der Kommunikation sieht er z.B. in der Struktur der medialen (Massen‑)Öffentlichkeit begründet.

10 Die Ansprachen und Regierungserklärungen sind Teil eines sich im Aufbau befindenden Korpus politischer Kommunikation, das derzeit an der Philipps-Universität Marburg in der AG Pragmalinguistik entsteht. Das Teilkorpus ›Corona-Diskurs‹ umfasst aktuell ca. 300 Texte, bestehend aus Ansprachen (in Form von TV-Ansprachen oder Podcasts und Facebook-Posts), Interviews, Talkshows, Regierungserklärungen, Pressekonferenzen, Pressestatements der Länder Schweiz, Österreich und Deutschland. Die Zusammensetzung der in diesem Beitrag untersuchten Daten wird im Literaturverzeichnis unter dem Punkt ‚Korpus‘ offengelegt.

11 Vgl. hierzu die Presseaussendungen unter https://www.bundeskanzleramt.gv.at/bundeskanzleramt/nachrichten-der-bundesregierung/2021.html (zuletzt aufgerufen am 19.05.2021) Die Pressestatements der einzelnen politischen Akteure in den Pressekonferenzen wurden von der Autorin für die Analyse verschriftlicht.

12 Vgl. hierzu die als Video eingestellten Pressekonferenzen unter https://www.bundeskanzlerin.de/bkin-de/mediathek/pressekonferenz-nach-dem-bund-laender-gespraech-am-22-maerz-1879994 (19.05.2021) Die Pressekonferenzen wurden von der Autorin für die Analyse verschriftlicht, sofern sie nicht im Wortlaut vorlagen.

13 Merkel (2020e).

14 Im Hinblick auf die Metaphernverwendung liegen auch Befunde vor, die aber aus Platzgründen in diesem Beitrag nicht thematisiert werden.

15 Je nach Fokus spielen in den Reden die Hochwertwörter *Demokratie, Freiheit, Solidarität, Hoffnung *und* Vertrauen* eine wichtige Rolle. Auf die Bedeutungsaushandlungen und Funktionen dieser Hochwertwörter kann im Rahmen dieser Analyse nur am Rande eingegangen werden. Zum Begriff der Solidarität in der Corona-Kommunikation vgl. auch Liedtke (2020).

16 Eine Ausmalung von Kriegsszenarien – wie sie beispielsweise Emmanuel Macron in seiner TV-Ansprache vom 15.3.2020 realisierte – bleibt bei Merkel und Kurz aus. Die Rede von Macron wird hier nicht untersucht. Vgl. dazu aber Spieß (2020). Eine Konzeptualisierung des Virus als kriegerischer Feind findet sich dennoch in den Ansprachen und stellt die Situation als gefährlich dar: »Also vorerst bleibt es dabei: Jeder und Jede ist ein Teil unseres **Kampfes gegen das Virus.**« (Merkel 2020b) oder bei Kurz: »Und auch, wenn **unser gemeinsamer Feind, das Virus**, nicht verschwinden wird, und wir die weltwirtschaftliche Situation als kleines Österreich kaum beeinflussen können […]« (Kurz 2020f) (Hervorhebungen CS).

17 Diese Aussage von Kurz in der Pressekonferenz vom 2.12.2020 wurde auch medial kontrovers diskutiert. Vgl. https://www.sueddeutsche.de/politik/kurz-migranten-suendenboecke-1.5136476 (1.4.2021).

18 Der Bezug auf den Freiheitsbegriff spielt in der Krisenkommunikation eine entscheidende Rolle zur Handlungsbegründung. Die Befürworter der pandemiebedingten Einschränkung formulieren die Wiedererlangung von Freiheit als Ziel oder heben auch hervor, dass die Freiheit des Einzelnen nur so weit gehen kann, wie die Freiheit des anderen nicht beschnitten wird. Deutlich wird das in der Aussage von Ralph Brinkhaus in der Debattenrede vom 29.10.2020: »Aber Freiheit ist nicht nur die Freiheit der Starken und Jungen. Freiheit ist auch die Freiheit der COPD-Patienten, die nicht aus dem Haus kommen.« Merkel beispielsweise bringt in der Regierungserklärung vom 29.10.2020 im Kontext der Maßnahmenbegründungen Freiheit mit Verantwortung in Verbindung (vgl. Merkel 2020h). Dagegen stellen die Kritiker der Maßnahmen diese Einschränkungen mit Bezug auf die Freiheitsrechte in Frage, so z.B. Alexander Gauland in seiner Debattenrede vom 29.10.2020. Die hier erwähnte Bundestagsdebatte vom 29.10.2020 kann eingesehen werden unter: https://dserver.bundestag.de/btp/19/19186.pdf (1.4.2021).

19 Kurz (2020a).

20 Vgl. für eine differenzierte Analyse der Modalverbensemantik im Kontext der Corona-Krisenkommunikation auch den Beitrag von Müller in diesem Heft.

21 Kurz (2020c) adressiert mit dieser Anrede am Ende seiner Ansprache nochmals explizit die Österreicherinnen und Österreicher.

22 In der Rede vom 15.3. wird in 8 von 18 Fällen mit der Wir-Verwendung auf die Regierung referiert.

23 Kurz (2020d).

24 Merkel (2020c).

25 Efing (2005) hat für Wahlkampfsituationen Strategien herausgearbeitet, die sich z.T. auch in den vorliegenden und untersuchten Reden finden lassen, obwohl es sich nicht um eine Wahlkampfsituation handelt.
